# Polymorphism of DNA repair genes in breast cancer

**DOI:** 10.18632/oncotarget.26568

**Published:** 2019-01-11

**Authors:** Beata Smolarz, Magdalena M. Michalska, Dariusz Samulak, Hanna Romanowicz, Luiza Wójcik

**Affiliations:** ^1^ Laboratory of Cancer Genetics, Department of Clinical Pathology, Polish Mothers' Memorial Hospital-Research Institute, Lodz, Poland; ^2^ Department of Obstetrics and Gynaecology, Regional Hospital in Kalisz, Poland; ^3^ Cathedral of Mother's and Child's Health, Poznań University of Medical Sciences, Poznań, Poland

**Keywords:** breast cancer, DNA repair, polymorphism, gene

## Abstract

**Aim:**

The aim of the study was to determine the relationship between single nucleotide polymorphisms (SNPs) of DNA repair genes and modulation of the risk of breast cancer. The following SNPs were analysed: XRCC1-Arg399Gln (rs25487), hMSH2-Gly322Asp (rs4987188), *XRCC2*-Arg188His (rs3218536), *XPD*- Lys751Gln (rs13181), *RAD51*--4719A/T (rs2619679) and *RAD51*--4601A/G (rs5030789).

**Material and Methods:**

The study included *n* = 600 patients: 300 with breast cancer and 300 healthy controls. The HRM (High-Resolution Melter) technique was applied for polymorphism analysis. Odds ratios (ORs) and 95% confidence intervals (CIs) were calculated for each genotype and allele.

**Results:**

Statistically significant correlations were identified between four single nucleotide polymorphisms and the breast cancer risk: *XRCC1*-Arg399Gln, *hMSH2*-Gly322Asp, *XPD*- Lys751Gln and *RAD51*--4719A/T. Allele XRCC1-Gln (OR 6.37; 95% CI 4.86–8.35, *p* < .0001), *hMSH2*-Asp (OR 4.41; 95% CI 3.43–5.67, *p* < .0001), XPD -Gln (OR 2.56; 95% CI 2.02–3.25, *p* < .0001) and *RAD51*-T genes (OR 1.44; 95% CI 1.15–1.80, *p* = 0.002) strongly correlated with breast carcinoma. No relationship was observed between the studied polymorphisms and the cancer progression grade according to Scarf-Bloom-Richardson classification.

**Conclusions:**

The results implies that polymorphisms of DNA repair genes may be associated with breast cancer occurrence.

## INTRODUCTION

Breast cancer is the most frequent malignancy among women. Almost 16,000 of women in Poland are diagnosed annually with breast cancer; the death toll is greater than 5,000. It is estimated that this annual morbidity may rise up to 20 thousand women in 2020 [1, 2]. Following the latest data of Eurocare-5, a five-year survival is achieved by 71.6% of female patients in Poland vs. 82% of the mean European value. Regarding breast cancer, the percent of successful 5-year treatment outcomes in Poland is 10% lower than the EU average [3].

Breast cancer is characterized by the occurrence of different genetic changes in various genes [4–6]. Consequently, it is often not possible to give a straightforward answer to the question, whether these changes are more like causes or more like effects of the disease. If they are perceive as causes, it is justified to study if the genetic variability, observed in many populations and defined as genetic polymorphism, may in any way contribute to induction and/or development of malignant changes, including breast cancer. Breast cancer is a disease with significant genetic component, characterized by high cure rates with early diagnosis [7–9]. This is the reason why prophylactic examinations of subjects from the group with high genetic risk would facilitate early diagnosis, eventually leading to reduced mortality rates [7–9]. DNA repair is a part of the barriers, protecting from cancer forming mutations [10–12]. Following the results of studies, cancer diseases are driven by a compromised ability of DNA repair. Therefore, a set of alleles of repair protein encoding genes may largely define the individual abilities for DNA damage repair, as well as the susceptibility to tumor development. It is then important to learn the polymorphic variants of the genes, associated with DNA repairs, as well as with their degradation in their population. The single nucleotide polymorphisms (SNPs) may change the risk of cancer. SNPs may then be regarded as potential markers of carcinogenesis [13, 14].

In our research projects, we concentrated on an evaluation of the role of single nucleotide polymorphisms of DNA repair genes in the pathogenesis of breast cancer and in the prognosis of its further course. The primary objectives of our studies were to identify the SNPs, associated with the risk of breast cancer in women of the Polish population, and to estimate the risk of their carrier-state. The results of our studies may help better understand the molecular background of the disease formation and enable to evaluate the probability of its occurrence in specific subjects of the population. The polymorphisms of DNA repair genes were selected on the basis of literature data, which are highly suggestive of their correlations with cancers development.

The alternations in DNA mismatch repair (MMR) genes have been associated with cancers development. *hMSH2* (MutS protein homolog 2) gene is involved in the MMR system [15]. Following data from the world literature, Gly322Asp polymorphism of *hMSH2* gene may enhance the risk of malignancy in the colon or the stomach, as well as increase the incidence rate of lymphomas, anaemia and breast cancer [16–22]. Breast cancer risk may be correlated with endogenous oestrogens [23]. Oestrogens induce DNA damage by producing reactive oxygen species during metabolic reactions [24]. The oestrogens may bring about oxidative DNA defects, which are eliminated by the base excision repair (BER) and nucleotide excision repair (NER) systems. X-ray repair cross-complementing 1 (*XRCC1*) gene is involved in the BER pathway [25]. The Arg399Gln polymorphism of *XRCC1* gene was selected on the basis of literature data, which are highly suggestive of its correlations with breast cancer development [26–28]. Oestrogens are genotoxic and influence carcinogenesis by the formation of bulky DNA adducts [29]. This type of damage is generally repaired by the nucleotide excision repair (NER) pathway. The *XPD* (the xeroderma pigmentosum group D) gene is involved in the NER pathway [30, 31]. The Lys751Gln (rs13181) polymorphism is one of the most widely studied genetic markers in *XPD* and its role in cancer development is evident [31]. Homologous recombination repair (HR) is also involved in the repair of DNA-protein cross-links in cooperation with NER. *XRCC2* and *RAD51* genes are key components of homologous recombination. According to literature data, the most frequently studied single nucleotide polymorphism of *XRCC2* gene is Arg188His. It has been demonstrated in a number of reports that it plays a significant role in the development of neoplastic diseases, including breast cancer [32–35]. Earlier reports of many researchers, dealing with SNPs in *RAD51* gene concentrated mainly on G135C and G172T polymorphisms at 5′ region, not subject of translation [36–40]. Our assumption was such that another genetic variability could act either additively or independently of the above-mentioned polymorphisms in 5′UTR region, what may help explain the role of *RAD51* in breast cancer development. Our research was then oriented towards less investigated SNPs within *RAD51* promoter gene: -4719A/T (rs2619679) and -4601A/G (rs5030789).

We examined 300 female patients with breast cancer and 300 healthy women for polymorphisms of the genes, belonging to: - the BER system *XRCC1* gene - Arg399Gln polymorphism (rs25487), - the NER system *XPD* gene - Lys751Gln polymorphism (rs13181), - the MMR system *hMSH2* gene - Gly322Asp (rs4987188), - the HR system *XRCC2* gene Arg188His (rs3218536), *RAD51* gene -4719A/T (rs2619679) and *RAD51* gene -4601A/G (rs5030789).

## RESULTS

In the studies on a series of 300 DNA samples from patients with breast cancer, originating from an ethnically homogenous population, we found a relationship of the studied polymorphisms with breast cancer occurrence (Table 1).

**Table 1:** Frequency distribution of the DNA repair genes genotypes/alleles in patients and controls, and the risk of breast cancer

	Patients (*n* = 300)		Controls (*n* = 300)			
XRCC1-Arg399Gln	Number	(%)	Number	(%)	OR (95% CI)^a^	*p*-value
Arg/Arg	34	11.33	66	22.00	1.00 Ref	
Arg/Gln	38	12.67	180	60.00	0.41 (0.23-0.70)	0.0017
Gln/Gln	228	76.00	54	18.00	8.19 (4.92-13.63)	<.0001
Arg	106	17.67	312	52.00	1.00 Ref	
Gln	494	82.33	288	48.00	6.37 (4.86-8.35)	<.0001
*hMSH2*-Gly322Asp	Number	(%)	Number	(%)	OR (95% CI)	*p*-value
Gly/Gly	42	14.00	80	26.67	1.00 Ref	
Gly/Asp	51	17.00	177	59.00	0.54 (0.33-0.89)	0.021
Asp/Asp	207	69.00	43	14.33	9.16 (5.57-15.07)	<.0001
Gly	135	22.5	337	56.17	1.00 Ref	
Asp	465	77.5	263	43.83	4.41 (3.43-5.67)	<.0001
*XPD*-Lys751Gln	Number	(%)	Number	(%)	OR (95% CI)	*p*-value
Lys/Lys	54	18.00	96	32.00	1.00 Ref	
Lys/Gln	70	23.33	120	40.00	1.03 (0.66-1.37)	1.000
Gln/Gln	176	58.67	84	28.00	3.72 (2.44-5.68)	<.0001
Lys	178	29.67	312	52.00	1.00 Ref	
Gln	422	70.33	288	48.00	2.56 (2.02-3.25)	<.0001
*XRCC2*-Arg188His	Number	(%)	Number	(%)	OR (95% CI)	*p*-value
Arg/Arg	72	24.00	80	26.67	1.00 Ref	
Arg/His	110	36.67	106	35.33	1.15 (0.76-1.74)	0.571
His/His	118	39.33	114	38.00	1.15 (0.76-1.73)	0.571
Arg	254	42.33	266	44.33	1.00 Ref	
His	346	57.67	334	55.67	1.08 (0.86-1.36)	0.521
*RAD51*-4719A/T	Number	(%)	Number	(%)	OR (95% CI)	*p*-value
A/A	60	20.00	75	25.00	1.00 Ref	
A/T	126	42.00	150	50.00	1.05 (0.69-1.58)	0.887
T/T	114	38.00	75	25.00	1.90 (1.21-2.97)	0.007
A	246	41.00	300	50.00	1.00 Ref	
T	354	59.00	300	50.00	1.44 (1.15-1.80)	0.002
*RAD51*-4601A/G	Number	(%)	Number	(%)	OR (95% CI)	*p*-value
A/A	78	26.00	87	29.00	1.00 Ref	
A/G	138	46.00	123	41.00	1.25 (0.84-1.84)	0.305
G/G	84	28.00	90	30.00	1.04 (0.67-1.59)	0.920
A	294	49.00	297	49.50	1.00 Ref	
G	306	51.00	303	50.50	1.02 (0.81-1.27)	0.920

^a^Crude odds ratio (OR), 95% CI = confidence interval at 95%.

This study demonstrated that *XRCC1*-Gln/Gln genotype of Arg399Gln polymorphism was strongly correlated with breast cancer. The Gln/Gln homozygote increased the risk of cancer (OR 8.19). The Gln allele in those patients may be a risk factor for breast cancer (OR 6.37; 95% CI 4.86–8.35, *p* < .0001).

The studies successfully demonstrated that *hMSH2*- Asp/Asp (OR 9.16; 95% CI 5.57–15.07, *p* < .0001), XPD-Gln/Gln (OR 3.72; 95% CI 2.44–5.68, *p* < .0001) and RAD51-T/T genotypes (OR 1.90; 95% CI 1.21–2.97, *p* = 0.007) were strongly associated with an increased risk of breast cancer.

In addition, the alleles of *hMSH2*-Asp (OR 4.41; 95% CI 3.43–5.67, *p* < .0001), *XPD* -Gln (OR 2.56; 95% CI 2.02–3.25, *p* < .0001) and *RAD51*-T genes (OR 1.44; 95% CI 1.15–1.80, *p* = 0.002) are strongly correlated with breast cancer.

No statistically significant differences were observed in genotype frequencies of *XRCC2* Arg188His polymorphism and *RAD51* -4601A/G polymorphism between the group of patients and healthy controls. (Table 3).

No relationship was observed between the studied polymorphisms and the cancer progression grade acc. to Scarf-Bloom-Richardson classification.

We did not find any correlation between the studies polymorphic variants of the repair genes and tumour grade or the lymph node status. Neither was there any relationship demonstrated between the analysed polymorphisms and the status of the oestrogen (ER), progesterone (PR) or HER2 receptors. DNA repair genes polymorphisms were also unrelated to the patients age, Hormone replacement therapy (HRT), BMI or menopause status.

## DISCUSSION

Our works fit in the general trend of studies, based on the commonly accepted and actual concept, assuming that the predisposition to cancers, including breast cancer, may be multigene in character, involving a relatively high number of low-penetration genes. In our studies, the groups of patients were ethnically uniform: female of the Polish origin, inhabitants of the Lodz Region.

We concentrated on an analysis of the relationship between Gly322Asp polymorphism of *hMSH2* gene and breast cancer. We selected the gene for its documented participation in the pathogenesis of cancers. Following data from the world literature, Gly322Asp polymorphism of *hMSH2* gene may enhance the risk of malignancy in the colon or the stomach, as well as increase the incidence rate of lymphomas, anaemia and breast cancer [41–43].

The Asp allele of Gly322Asp polymorphism was strongly correlated with breast cancer. The Gly322Asp polymorphism affected the tumour grade and size, as well as was associated with the lymph node status.

As literature data demonstrate DNA damages to be highly significant in the pathogenesis of breast cancer, especially those which require repair by homologous recombination [44, 45].

Therefore, our studies were continued in an analysis of subsequent polymorphisms in the gene, which encoded the protein, participating in repair by homologous recombination (*RAD51*).

We demonstrated a possible correlation of -4719A/T (rs2619679) and -4601A/G (rs5030789) polymorphisms of *RAD51* repair gene with breast cancer. Earlier reports of many researchers, dealing with SNPs in *RAD51* gene concentrated mainly on G135C and G172T polymorphisms at 5′ region, not subject of translation [46–49].

Since RAD51 participates in DNA repair, while also interacting with BRCA proteins, the mutations of which are often identified in breast cancer, the above-mentioned polymorphisms may be associated with a higher risk of this cancer development [50, 51].

It has been found, among others, that 135C variant may increase the risk of breast cancer in carriers of mutations in *BRCA1* and *BRCA2* genes, while no effects of 135C variant were observed on the morbidity level in women without the mutations. G135C polymorphism can modify the way of mRNA splicing, what, in turn, affects the protein functions or the effectiveness of translation [52].

Despite the abundance of results, there is still no unequivocal explanation of the role of RAD51 in cancer formation. Our assumption was such that another genetic variability could act either additively or independently of the above-mentioned polymorphisms in 5′UTR region, what may help explain the role of RAD51 in breast cancer development [46, 53, 54].

Our research was then oriented towards less investigated SNPs within *RAD51* promoter gene: -4719A/T (rs2619679) and -4601A/G (rs5030789). We describe a group of 300 female patients with breast cancer, demonstrating that TT genotype of -4719A/T polymorphism and GG genotype of -4601A/G polymorphism of *RAD51* gene was strongly correlated with breast cancer. Moreover, the AG heterozygote of -4601A/G polymorphism of *RAD51* gene was more frequently observed in the first grade of the cancer disease (acc. to Scarf-Bloom-Richardson) than in stage II and III. None of the studied polymorphisms were associated with tumour size or the lymph node status.

The presented studies demonstrated that *XRCC1*–Gln/Gln and *XPD*-Gln/Gln genotypes were strongly associated with an increased risk of breast cancer.

We did not find any correlation between the studies polymorphic variants of the repair genes and tumour grade or tumour size or the lymph node status. Neither did we show any relationship of the analysed polymorphisms with the age of the patients, BMI, hormone replacement therapy or menopausal status.

In summation, the above presented studies contribute to a better knowledge of the molecular background of breast cancer. The results indicate DNA repair genes and their polymorphisms can be involved in the breast cancer formation process in the population of women in Poland. They may find practical application to improve the early diagnostics of cancer and thus to extend the survival rates of women with breast cancer.

## MATERIALS AND METHODS

### Patients

Blood samples were obtained from women with breast carcinoma (*n* = 300), treated at the Department of Department of Oncological Surgery and Breast Diseases, Institute of Polish Mothers Memorial Hospital. Blood specimens were collected from the patients by venipuncture before treatment. The criteria for patient participation in this study were as follows: histologically confirmed diagnosis of breast carcinoma, primary surgical resection of the breast without receiving prior immuno-, radio- or chemotherapy, absence of distant metastases. All the diagnosed tumors were graded by criteria of Scarf-Bloom-Richardson. The age of the patients ranged in from 48 to 84 years (the mean age 57.2 ± 10.12). 300 age-matched disease-free women were selected as controls (age range 49–80, mean age 55.26 ± 10.17). The full characteristics of the study group are presented in Table 2. The Local Ethic Committee approved the study and each patient gave a written consent (Approval number, 10/2012).

**Table 2:** The characteristics of breast cancer cases (*n* = 300) and controls (*n* = 300)

	Patients (*n*, %)	Controls (*n*, %)
Premenopausal	112 (37.33)	98 (32.67)
Postmenopausal	188 (62.67)	202 (67.33)
Body mass index (BMI ≥ 30 kg/m2)		
Yes	140 (46.67)	100 (33.33)
No	160 (53.33)	200 (66.67)
Hormone replacement therapy (HRT)		
Never	114 (38.00)	120 (40.00)
Estrogen	186 (62.00)	180 (60.00)
Hormone receptor status		
ER+	72 (24.00)	
PR+	79 (26.33)	
HER2+	110 (36.67)	
ER+PR+HER2+	19 (6.33)	
ER-PR-HER2	20 (6.67)	
Histopathological grading		
G1	90 (30.00)	
G2	110 (37.00)	
G3	100 (33.00)	
Tumor size grade		
T1	75 (25.00)	
T2	135 (45.00)	
T3	90 (30.00)	
Lymph node status		
N0	90 (30.00)	
N1	85 (28.33)	
N2	75 (25.00)	
N3	50 (16.67)	

### DNA isolation and genotyping

Genomic DNA was prepared using QIAamp DNA Kit (Qiagen GmbH, Hilden, Germany) according to the manufacturer instruction. Real-time PCR cycling and conditions and primers for HRM analysis of all the examined DNA repair SNPs are summarized in Table 3. CR amplification was performed with support of a Light Cycler^®^ 480 High Resolution Melting Master Kit (Roche, Mannheim, Germany), according to the manufacturer's recommendations. The HRM technique was carried out in a LightCycler^®^ 96 (Roche, Mannheim, Germany) Thermocycler. A non-template control contained water, instead of genomic DNA, as a negative control. Additionally, positive controls (DNA samples with known genotype) were employed in each run of HRM analysis. All the control DNA samples were employed in each run of HRM analysis. The collected data were analyzed, using the LightCycler^®^ 96 software version SW 1.1 (Roche, Mannheim, Germany). SNPs in DNA repair genes were selected using the public domain of the National Center for Biotechnology Information at http://www.ncbi.nlm.nih.gov/snp (Bethesda, MD, USA). Primer3 software (http://frodo.wi.mit.edu/, Tartu, Estonia) was used for primers design.

**Table 3:** The refSNP and thermal conditions for HRM analysis

	Polymorphism	refSNP	Primer sequence F-forward, R-reverse	Thermal conditions
*XRCC1*	Arg399Gln	rs25487	F 5′-TAAGGAGTGGGTGCTGGACT-3′ R 5′-ATTGCCCAGCACAGGATAAG-3′	PCR cycling (40 cycles) Denaturation 30 s for 95° C Annealing 30 s for 58° C Extension 30 s for 72° C HRM 75–90° C
*hMSH2*	Gly322Asp	rs4987188	F 5′-GTTTTCACTAATGAGCTTGC-3′ R 5′-AGTGGTATAATCATGTGGGT-3′	PCR cycling (40 cycles) Denaturation 30 s for 95° C Annealing 30 s for 55° C Extension 30 s for 72° C HRM 75–90° C
*XPD*	Lys751Gln	rs13181	F 5′-CCTCTGTTCTCTGCAGGAGGA-3′ R 5′-CCTGCGATTAAAGGCTGTGGA-3′	PCR cycling (40 cycles) Denaturation 30 s for 95° C Annealing 30 s for 55° C Extension 30 s for 72° C HRM 75–90° C
*XRCC2*	Arg188His	rs3218536	F 5′-TGTAGTCACCCATCTCTCTGC-3′ R 5′-AGTTGCTGCCATGCCTTACA-3′	PCR cycling (40 cycles) Denaturation 30 s for 95° C Annealing 30 s for 56° C Extension 30 s for 72° C HRM 75–90° C
*RAD51*	-4719A/T	rs2619679	F 5′-AGATAAACCTGGCCAACGTG-3′ R 5′-CCGTGCAGGCCTTATATGAT-3′	PCR cycling (40 cycles) Denaturation 30 s for 95° C Annealing 30 s for 57° C Extension 30 s for 72° C HRM 75–90° C
*RAD51*	-4601A/G	rs5030789	F 5′-AGATAAACCTGGCCAACGTG-3′ R 5′-CCGTGCAGGCCTTATATGAT-3′	PCR cycling (40 cycles) Denaturation 30 s for 95° C Annealing 30 s for 58° C Extension 30 s for 72° C HRM 75–90° C

### Statistical analysis

Genotype and allele distributions were compared with those expected for a population in Hardy-Weinberg equilibrium (HWE) by using the Chi-square test. Genotype and allele frequencies in cases and controls were compared by χ^2^-test. Genotype and allele evaluation, regarding their relationship with a given feature, e.g., a risk for disease, was supported by an analysis of odds ratio (ORs) and 95% confidence intervals, calculated according to the logistic regression model. The wild type of genotype and allele was a reference group. *P*-values < 0.05 were considered significant.

## Abbreviations

CI: Confidence interval
HRM: High resolution melting
HR: Homologous recombination repair
HWE: Hardy-Weinberg equilibrium
BER: Base excision repair
NER: Nucleotide excision repair
MMR: Mismatch repair
OR: Odds ratio
SNP: Single nucleotide polymorphism
